# Acetylation enables a dual-targeting fungal effector to inhibit insect immunity and development

**DOI:** 10.1371/journal.ppat.1014680

**Published:** 2026-10-05

**Authors:** Yulong Wang, Ying Dong, Yang Zhang, Siying Wu, Deshui Yu, Guang Yang, Shuqian Gao, Yang Yang, Shihao Dong, Li Qin, Bo Huang

**Affiliations:** 1 Anhui Provincial Key Laboratory of Biological Control, Anhui Agricultural University, Hefei, China; 2 Hefei Forestry Protection Center, Hefei, China; Chinese Academy of Sciences, CHINA

## Abstract

Pathogenic microbes deploy secreted effectors to subvert host immunity and physiology across kingdom boundaries, yet the molecular mechanisms that maintain effector competence during this transition remain poorly defined. Here, we identify MrNIS1, an effector from the insect-pathogenic fungus *Metarhizium robertsii* whose stability and secretion are governed by K24 acetylation through physical association with the acetyltransferase MrKAT1 and the deacetylase MrSIR2. Once inside the insect, MrNIS1 acts as a dual-targeting effector to disrupt distinct physiological pathways. It binds the serine protease SPS to block prophenoloxidase activation, thereby suppressing melanization-based immunity. MrNIS1 interacts with the evolutionarily conserved cholesterol transporter ERP, disrupting ecdysteroid biosynthesis and leading to impaired larval growth and cuticle synthesis. This MrNIS1-ERP interaction is functionally conserved across Lepidoptera, Coleoptera, and Diptera. Furthermore, heterologous expression in *Beauveria bassiana* demonstrates that the KAT1/SIR2 regulatory system is conserved across fungal species, where MrNIS1 acts as a portable virulence module to significantly enhance pathogen efficacy. Our findings reveal that lysine acetylation serves as a molecular switch that preserves effector competence during cross-kingdom transit, enabling the simultaneous targeting of multiple host physiological processes. These results elucidate a conserved strategy of fungal pathogenesis and identify potential targets for the improvement of microbial biocontrol agents.

## Author summary

Fungal pathogens secrete effectors to manipulate hosts, but how these weapons remain stable during transmission is unclear. Our study reveals that lysine acetylation of an insect-pathogenic fungal effector ensures its stability, delivery, and function inside hosts. Once there, it simultaneously disrupts immune defense and hormone signaling—blocking both protective melanization and larval development. This regulatory system can be transferred to other fungi to enhance their virulence. These findings uncover a molecular mechanism preserving effector competence across kingdoms and offer a strategy to improve biological pest control.

## Introduction

Species of the entomopathogenic genus *Metarhizium* are major biological control agents used for the sustainable management of agricultural and forest pests. Their efficacy as biocontrol agents depends on specialized molecular strategies that allow them to bypass or suppress host immune defenses. Mycopathogens secrete a pleiotropic effector arsenal that collectively disable host immunity, thereby opening the gateway for promoting infestation, expansion, and systemic colonization [1,2]. Phytopathogenic and entomopathogenic microbes extend this strategy, injecting or translocating proteins, small RNAs or secondary metabolites into host cells [3]. Once delivered, these effectors mask microbial identity, jam pattern-recognition receptors, re-wire hormone and signalling networks, subvert ubiquitin proteasome and autophagy machineries, neutralise antimicrobial peptides and reactive oxygen species (ROS), and reprogram transcriptional, RNA-silencing and cell-death circuits [4–6]. Through these concerted manipulations, pathogens sculpt a permissive ecological niche that fuels their proliferation and kingdom-spanning dissemination [7]. Therefore, dissecting the effector repertoire of mycopathogens promises to yield pivotal insights for devising novel and effective strategies to prevent and control these devastating pests.

Host–pathogenic co-evolution operates as an escalating molecular arms race wherein antagonistic selection on fungal effector loci catalyzes iterative post-translational modifications (PTMs), enabling stepwise evasion of host immune surveillance and successive redefinition of the virulence–resistance interface [8]. For instance, 39 pathogenesis-related effectors were modified by redox modification in the rice blast fungus *Magnaporthe oryzae*, suggesting that redox modification could regulate functions of effector proteins for invasion in host cells [9]. Acetylation at Lys167 shields the *Fusarium oxysporum* effector FolSpv1 from ubiquitin-mediated proteolysis, enabling its stable accumulation and subsequent translocation of tomato SlPR1 to achieve full virulence in host tissues [10]. While most studies have centered on effector-catalyzed PTMs of host proteins, the functional impact of PTMs that occur on the effectors themselves remains largely unexplored [8,11–13].

Necrosis-inducing secreted protein 1 (NIS1) is a phylogenetically conserved effector that spans the Ascomycota and Basidiomycota [14]. First characterized in *Colletotrichum orbiculare*, NIS1 disables plant pattern-triggered immunity by docking onto and catalytically silencing the receptor-like kinases BAK1 and BIK1, thereby blocking ROS bursts and downstream defense gene activation [15]. A C-terminal extension of the same protein can trigger necrosis in *Nicotiana benthamiana*, demonstrating that its immune-suppressive and cell-death-inducing activities are genetically separable [16]. Beyond plant pathosystems, orthologs in entomopathogenic fungi such as *Metarhizium robertsii* neutralize insect immunity by targeting the hemocyte defense protein Hdd11, underscoring a conserved, kingdom-spanning strategy of host disarmament [17]. Consequently, despite its central role in virulence on crops and Lepidopteran pests, the molecular and physiological repertoire of this ancient effector remains tantalizingly unresolved.

Previously, we identified two NIS1 homologs in *M. robertsii* and demonstrated that MrNLS1 suppresses lepidopteran immunity by targeting the host defense protein Hdd11 [17]. In this study, we report that the second homolog, MrNIS1, is regulated by a reversible acetylation switch at Lysine 24 (K^24^). We show that this modification is required for MrNIS1 stability and its subsequent secretion into the host hemolymph. Once translocated, MrNIS1 functions as a dual-targeting effector that simultaneously inhibits the serine protease SPS to block PO-mediated melanization and interacts with the conserved cholesterol transporter ERP to disrupt ecdysteroid biosynthesis and larval growth. The functionality of this system is further evidenced by its successful transfer to *B. bassiana*, where MrNIS1 is similarly acetylated and significantly enhances virulence. These findings identify lysine acetylation as a critical molecular requirement for coordinating multi-target effector strategies during inter-kingdom fungal pathogenesis.

## Results

### Acetylated MrNIS1 interacts with MrKAT1 and MrSIR2

Our previous genome-wide yeast two-hybrid (Y2H) screen using the deacetylase MrSIR2 as bait identified MrNIS1 among 24 candidate interacting proteins, suggesting that MrNIS1 may undergo lysine acetylation [18]. To systematically identify the enzymatic machinery governing this modification, we conducted a genome-wide Y2H screen using MrNIS1 as bait. This screen identified 12 candidate interactors, including the acetyltransferase MrKAT1 and the deacetylase MrSIR2 (Table A in S1 Appendix). Targeted Y2H and pull-down assays confirmed that both MrKAT1 and MrSIR2 physically associate with MrNIS1 (Fig 1A and 1B). To verify the acetylation status *in vivo*, we performed an acetylome analysis of *M. robertsii* mycelia. Peptides corresponding to MrNIS1 were successfully enriched using anti-acetyllysine agarose beads, as confirmed by mass spectrometry (Fig 1C).

**Fig 1:**
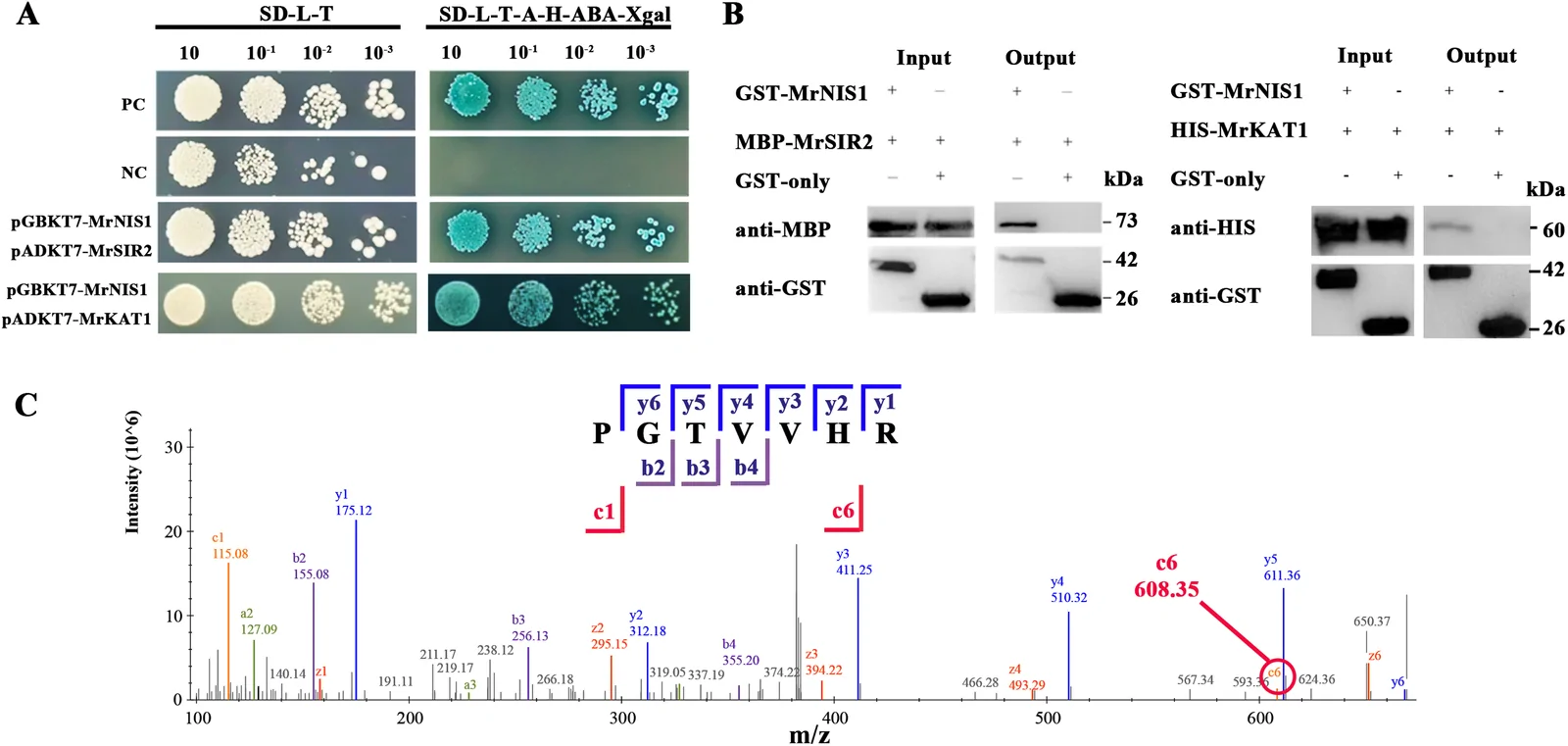
MrNIS1 acetylation modified by MrSIR2 and MrKAT1. (A) Yeast two-hybrid assays showing the interactions of MrNIS1 with the deacetylase MrSIR2 and the acetyltransferase MrKAT1. (B) GST pull-down assays showing the interaction of MrSIR2 and MrKAT1 with MrNIS1 *in vitro*. (C) Representative tandem mass spectra showing the acetylation of MrNIS1.

### K24 acetylation is essential for MrNIS1 vacuolar accumulation and secretion

MrNIS1 possesses two lysine residues, K24 and K120 (Fig 2A). To map the specific acetylation site, we generated substitution mutants (Lysine to Arginine) tagged with GFP. Immunoblotting of GFP immunoprecipitates with an anti-acetylated lysine antibody revealed that while the K120 mutation did not affect acetylation levels, substitution of K24 completely abolished MrNIS1 acetylation, identifying K24 as the primary acetylation site (Fig 2B).

**Fig 2:**
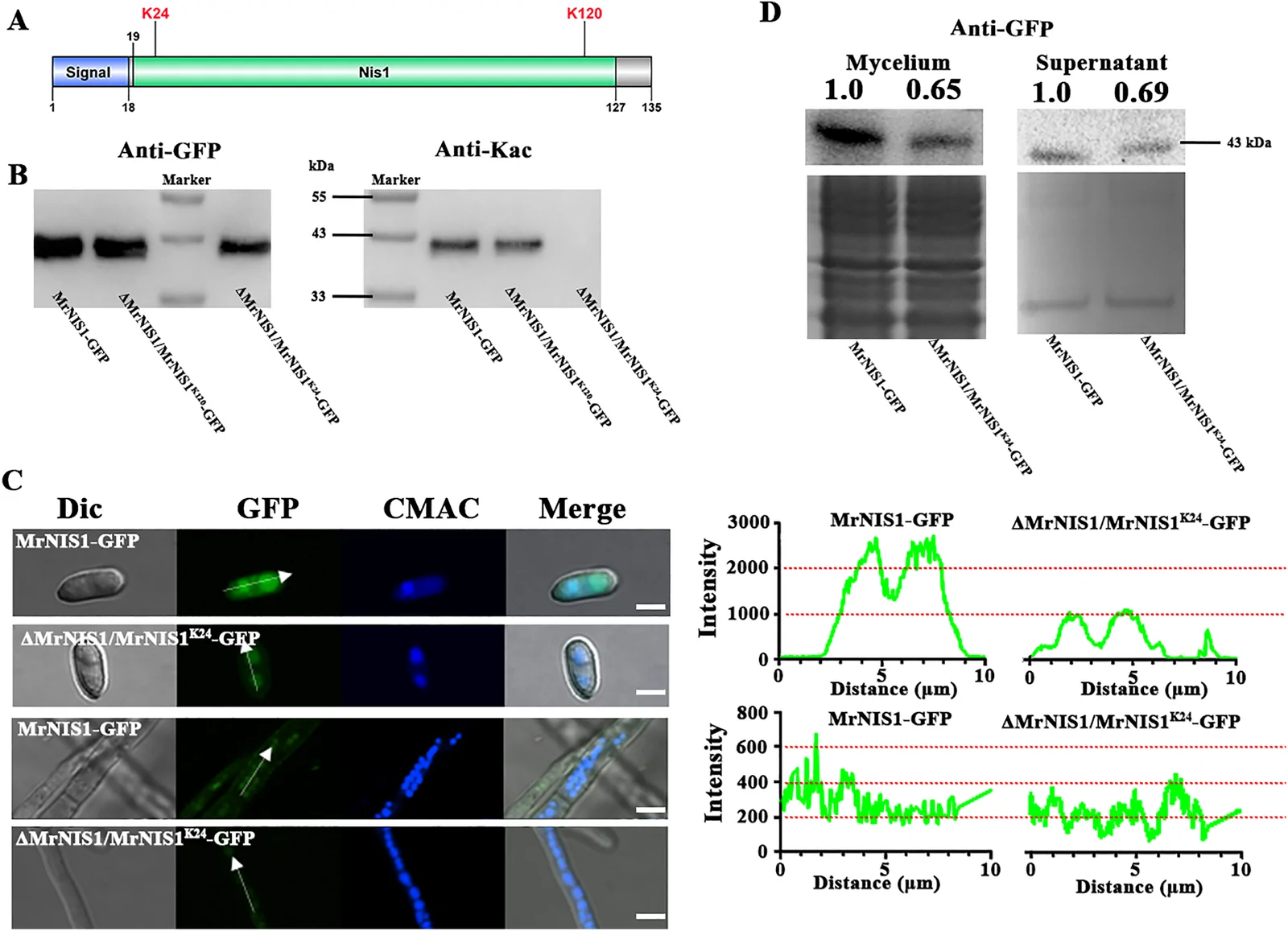
Lysine 24 acetylation is essential for MrNIS1 stability and secretion. (A) Schematic representation of MrNIS1 showing the locations of two lysine residues (K24 and K120). (B) Immunoprecipitation of GFP-tagged MrNIS1 variants followed by immunoblotting with anti-acetyl-lysine antibody. (C) Fluorescence microscopy of complemented strains expressing MrNIS1-GFP or MrNIS1^K24^-GFP in the ΔMrNIS1 background. Scale bar: 2 μm. (D) Western blot analysis of MrNIS1-GFP and MrNIS1^K24^-GFP in mycelial extracts (left) and culture supernatants (right). Coomassie blue staining of parallel gels served as loading controls.

Confocal microscopy analysis revealed that MrNIS1 localizes to fungal vacuoles in both conidia and hyphae (Fig 2C). GFP fluorescence was markedly reduced in both conidia and mycelia of the K24 mutant compared to wild-type. Consistent with this observation, immunoblot analysis of mycelial extracts showed substantially decreased protein levels of MrNIS1^K24^-GFP relative to MrNIS1-GFP (Fig 2D). Previous studies have established NIS1 as a conserved secreted effector in filamentous fungi. SignalP-5.0 prediction identified a putative N-terminal signal peptide in MrNIS1 (amino acids 1–18), suggesting extracellular secretion [14]. To assess the role of K24 acetylation in secretion, we examined MrNIS1 levels in culture supernatants. The K24 mutation resulted in reduced secretion of MrNIS1 into the extracellular medium (Fig 2D).

### K24 acetylation preserves MrNIS1 stability within the insect hemocoel

To determine the contribution of MrNIS1 to fungal virulence, we first examined its expression profile during *M. robertsii* infection of *Galleria mellonella* larvae. *MrNIS1* transcript levels peaked at 2–3 days post-infection, coinciding with hemocoel colonization, which suggests an important role during this stage (Figure A in S1 Appendix). We therefore generated an MrNIS1 knockout (*∆*MrNIS1) mutant. The mutant exhibited attenuated virulence against *G. mellonella* larvae, a phenotype that was fully rescued by WT complementation (cp∆MrNIS1) (Fig 3A and Table B in S1 Appendix). Complementation with the K24R mutant (∆MrNIS1/MrNIS1^K24^) failed to restore full virulence, whereas the K120R mutant (∆MrNIS1/MrNIS1^K120^) displayed WT-like pathogenicity.

**Fig 3:**
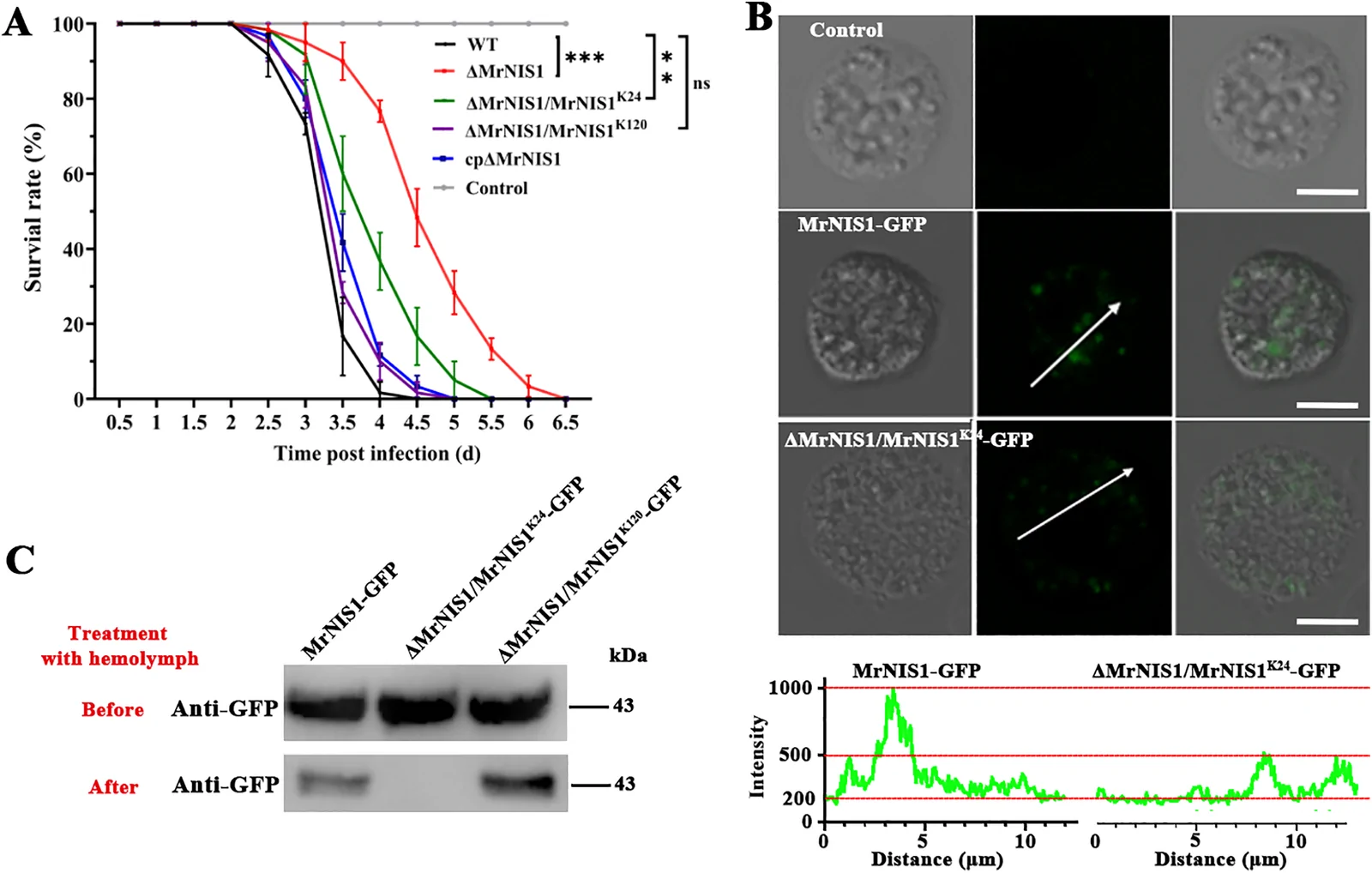
K24 acetylation is essential for MrNIS1 stability in the insect host. (A) Survival curves of *Galleria mellonella* larvae infected by different mutants. ^**^P < 0.01, ^***^P < 0.001. (B) Fluorescence microscopy of MrNIS1-GFP and MrNIS1^K24^-GFP localization in hemocytes from larval hemolymphs. Scale bar: 10 μm. (C) *Ex vivo* acetylation stability analysis. Purified proteins were incubated with insect hemolymph for 4 h and analyzed by immunoblotting with anti-GFP antibody.

To determine virulence contribution of the K24 acetylation, subcellular localizations of MrNIS1-GFP and MrNIS1^K24^-GFP in host were explored during the fungal infection (Fig 3B). Although GFP signals were observed in the hemocytes from the hemolymphs of insect infected both ∆MrNIS1/MrNIS1^K24^-GFP and MrNIS1-GFP, the K24 mutant displayed dramatically weaker fluorescence intensity compared with the MrNIS1-GFP. To explore the function of K24 acetylation of MrNIS1 in the insect environment, we purified MrNIS1^K24^-GFP, MrNIS1^K120^-GFP and MrNIS1-GFP from fungal mycelia using anti-GFP agarose beads, and incubated the proteins with insect hemolymph (Fig 3C). Although both MrNIS1-GFP and MrNIS1^K120^-GFP showed reduced abundance following treatment, MrNIS1^K24^-GFP was nearly undetectable. K24 acetylation thus safeguards MrNIS1 against host-derived proteolysis, maintaining its stability within the insect environment. As the K24R mutant mimics the deacetylated state, its rapid degradation in the host hemolymph indicates that K24 acetylation must be maintained for the native effector to remain stable after translocation into the host.

### MrNIS1 functions as a dual-targeting effector against host SPS and ERP

To investigate the function of MrNIS1 in the host, MrNIS1 without the SP as a bait to screen the *G. mellonella* cDNA library via Y2H system and 5 MrNIS1-interacting protein candidates were identified (Table C in S1 Appendix). Among them, serine protease snake-like (GmSPS) and ecdysteroid-regulated 16 kDa protein (GmERP) were identified as full-length clones containing complete coding sequences and selected for further research. Further Y2H and glutathione S-transferase (GST) pull-down assays analyses proved that GmSPS and GmERP interact with MrNIS1 (Fig 4A and 4B).

**Fig 4:**
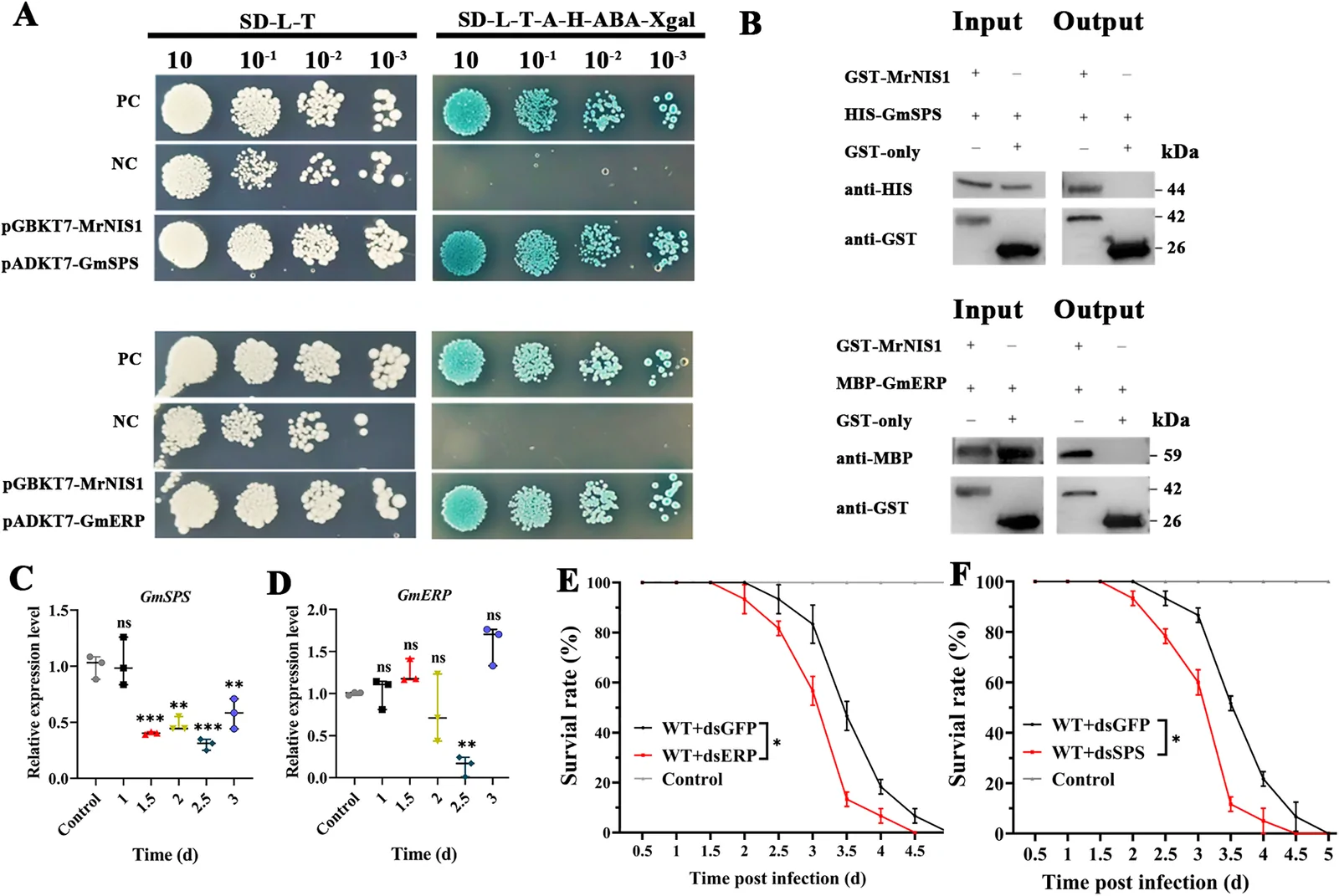
MrNIS1 interacts with insect SPS and ERP proteins. (A) Yeast two-hybrid assays showing the interactions of MrNIS1 with GmSPS and GmERP. (B) GST pull-down assays demonstrating the *in vitro* interactions of MrNIS1 with GmSPS (upper) and GmERP (lower). (C and D) Expression characteristics of *GmSPS* (C) and *GmERP* (D) in *G. mellonella* following topical infection with *M. robertsii*. (E and F) Survival curves of *G. mellonella* larvae challenged with *M. robertsii* after RNA interference targeting *GmERP* (E) and *GmSPS* (F). ^*^P < 0.05, ^**^P < 0.01, ^***^P < 0.001.

We next examined the expression of *GmSPS* and *GmERP* in *G. mellonella* after topical fungal infection for 1–3 days. *GmSPS* expression rapidly decreased following fungal entry into the hemocoel, and *GmERP* was markedly reduced at 2.5 days post fungal infection (Fig 4C and 4D). We employed RNA interference (RNAi) to knockdown *GmSPS* and *GmERP* expression in larvae (Figure B in S1 Appendix). Compared with control insects, *GmSPS* or *GmERP* knockdown insects exhibited marked reductions in immunity against *M. robertsii* (Fig 4E and Table B in S1 Appendix).

### MrNIS1 suppresses melanization through competitive inhibition of the GmSPS-PPO axis

Serine protease cascades play a pivotal role in the activation of prophenoloxidase (PPO) into active phenoloxidase (PO), thereby triggering the melanization immune response in insects [19]. To investigate whether GmSPS participates in the insect melanization cascade, a Y2H screen using pGBKT7-GmSPS as bait was performed and 17 potential interacting proteins, including phenoloxidase subunit 1 (GmPPO), were identified (Table D in S1 Appendix). The specific interaction between GmSPS and GmPPO was subsequently validated by directed Y2H assays and HIS pull-down experiments (Fig 5A).

**Fig 5:**
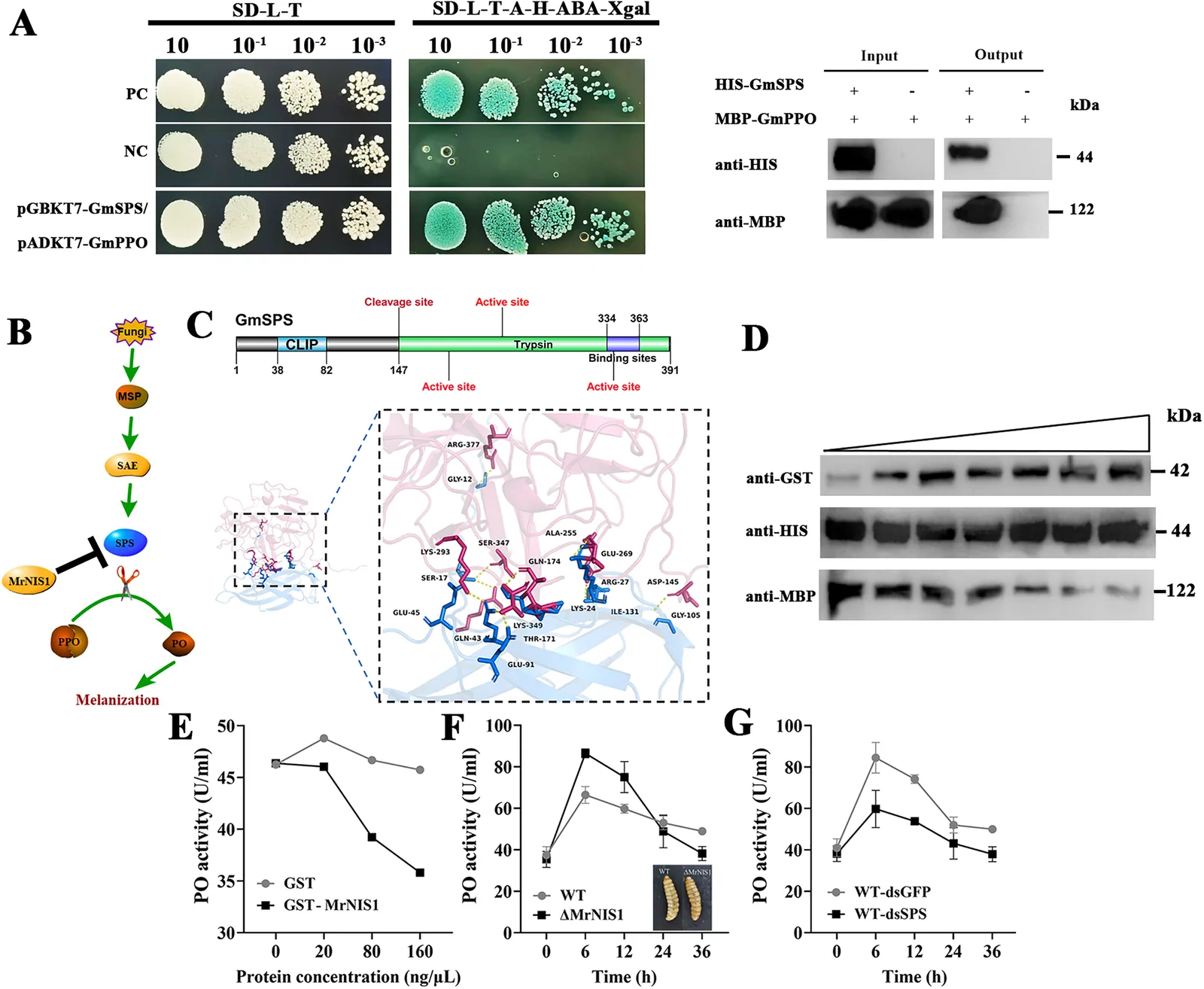
MrNIS1 inhibits PO activity by targeting insect SPS. (A) Yeast two-hybrid (left) and GST pull-down (right) assays demonstrating interaction between GmSPS and GmPPO. (B) Schematic diagram illustrating the proposed mechanism of MrNIS1 in modulating insect melanization immune response. (C) Predicted structure of the GmSPS-MrNIS1 complex generated by ZDOCK server. GmSPS is shown in red and MrNIS1 in blue. (D) Competitive binding among MrNIS1, GmSPS and GmPPO determined by competitive pull-down assay. HIS-GmSPS was immobilized on Ni-NTA resin and incubated with increasing concentrations of GST-MrNIS1 in the presence of a constant amount of MBP-GmPPO. (E–G) PO activity in hemolymph of larvae after injection with GST-MrNIS1 (E), topical application of ΔMrNIS1 (F) or injection with dsSPS. Data are presented as mean ± SD from three independent experiments (n = 6 per group).

To elucidate the functional consequence of the MrNIS1-GmSPS-GmPPO interaction, we hypothesized that GmSPS may function as a protease that cleaves the zymogen GmPPO into its active form, and MrNIS1 competitively bind to GmSPS, potentially obstructing the interaction between GmSPS and GmPPO, thereby preventing the proteolytic activation of GmPPO (Fig 5B). To gain mechanistic insights into this competitive inhibition, we performed protein-protein docking analysis to model the binding interface between GmSPS and MrNIS1 (Fig 5C and Figure C in S1 Appendix). The structural prediction revealed that multiple sites (D145, A255, E269, K293, S347, K349 and R377) within the trypsin domain of GmSPS were occupied upon MrNIS1 binding. Trypsin domain of serine protease can precisely cleave PPO to produce PO, suggesting that MrNIS1 binding may prevent GmSPS from interacting with GmPPO [19].

To validate the competitive binding hypothesis, we conducted *in vitro* competitive pull-down assays. Increasing concentrations of GST-MrNIS1 were incubated with a fixed amount of His-GmSPS, followed by the addition of MBP-GmPPO. The results demonstrated a dose-dependent decrease in the amount of MBP-GmPPO that co-precipitated with His-GmSPS as the concentration of GST-MrNIS1 increased (Fig 5D).

To assess the physiological relevance of this competitive inhibition, we performed *in vivo* phenoloxidase activity assays following direct injection of recombinant GST-MrNIS1 into the hemocoel of insect larvae. As shown in Fig 5E, PO activity in the hemolymph was reduced in a dose-dependent manner following injection of GST-MrNIS1, with higher concentrations resulting in more pronounced suppression of PO activity.

To further substantiate the role of MrNIS1 in suppressing PO activation during fungal infection, we compared PO activity in larvae infected with wild-type *M. robertsii* versus ΔMrNIS1. Larvae infected with the ΔMrNIS1 mutant displayed markedly elevated PO activity in the hemolymph throughout the first 24 hours post-infection relative to those challenged with the wild-type strain. At 24 hours post-infection, insects infected with the ΔMrNIS1 mutant exhibited a darker external coloration compared to those infected with the wild-type strain, indicative of enhanced melanization (Fig 5F).

Finally, to confirm the essential role of GmSPS in PPO activation, RNAi was performed for knockdown of GmSPS expression in larvae. Depletion of GmSPS resulted in a significant reduction in PO activity in the hemolymph compared to control larvae, demonstrating that GmSPS is indeed required for the proteolytic activation of PPO (Fig 5G). Collectively, these findings establish a molecular mechanism whereby the fungal effector MrNIS1 targets the insect serine protease GmSPS through competitive binding, thereby inhibiting GmSPS-mediated cleavage of GmPPO and suppressing the melanization immune response.

### MrNIS1 targets GmERP to impair larval development and cuticle synthesis

ERP, characterized by its Niemann-Pick type C-2 (NPC2) domain with conserved cholesterol/lipid-binding sites, participates in insect growth and cuticle synthesis [20,21]. To elucidate the functional significance of the interaction between MrNIS1 and GmERP, we first investigated the effect of MrNIS1 on *G. mellonella* larval development. Recombinant GST-MrNIS1 was injected into the hemocoel of larvae, resulted in a significant reduction in both larval body length and weight compared to control larvae injected with GST alone (Fig 6A and 6B). Depletion of GmERP led to a marked decrease in larval growth rate, phenocopying the effect observed upon MrNIS1 injection (Fig 6C).

**Fig 6:**
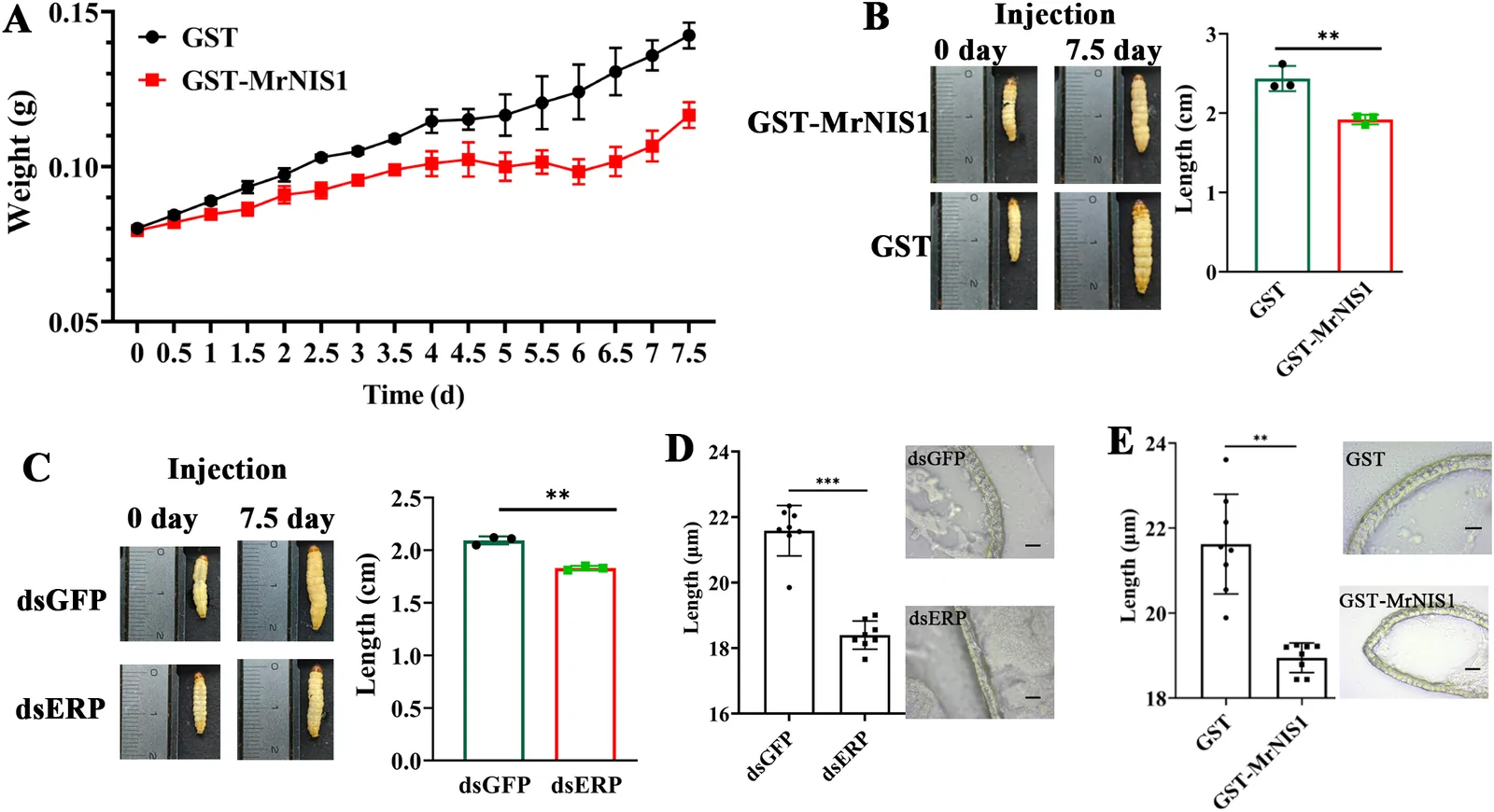
SPS is essential for insect growth and cuticle development. (A and B) Changes in larval weight (A) and body length (B) of 2-day-old third-instar larvae after injection with 1 μg of GST-MrNIS1 (n = 15 per group). (C) Larval length change of 2-day-old third-instar larvae after RNAi-mediated knockdown of *GmERP* (dsERP). Data are presented as mean ± SD from three independent experiments (n = 6 per group). (D and E) Cuticle thickness in larvae treated with dsERP (D) or GST-MrNIS1 (E). Eight randomly selected larvae were sectioned and measured. Data are presented as mean ± SD. Scale bar, 20 μm. ^**^P < 0.01, ^***^P < 0.001.

Given the established role of ERP/NPC2 proteins in cholesterol trafficking and ecdysteroid biosynthesis as mentioned above, we hypothesized that disruption of GmERP function might affect cuticle formation, and measured the cuticle thickness of larvae following GmERP knockdown. RNAi-mediated depletion of GmERP for 7.5 days resulted in a significant reduction in cuticle thickness compared to control larvae, demonstrating that GmERP is required for normal cuticle synthesis and integrity (Fig 6D). Consistent with the GmERP knockdown phenotype, MrNIS1-injected larvae exhibited reduced cuticle thickness at 7.5 days post-injection compared to control larvae (Fig 6E).

### Functional conservation of the MrNIS1-ERP interaction across insect orders

Phylogenetic analysis revealed that ERP homologs are highly conserved among major insect orders, including Lepidoptera, Diptera, Coleoptera, and Orthoptera, all containing the characteristic NPC2 domain, suggesting functional conservation of ERP-mediated physiological processes throughout insect evolution (Fig 7A). Based on this sequence conservation, we hypothesized that MrNIS1 might possess the capacity to interact with ERP proteins from divergent insect species. Yeast two-hybrid assays demonstrated that MrNIS1 indeed directly binds to ERP proteins from the *Bombyx mori*, *Drosophila melanogaster*, *Molorchus minor*, and *Locusta migratoria*, indicating that the MrNIS1-ERP interaction interface is conserved across phylogenetically distant insect hosts (Fig 7B).

**Fig 7:**
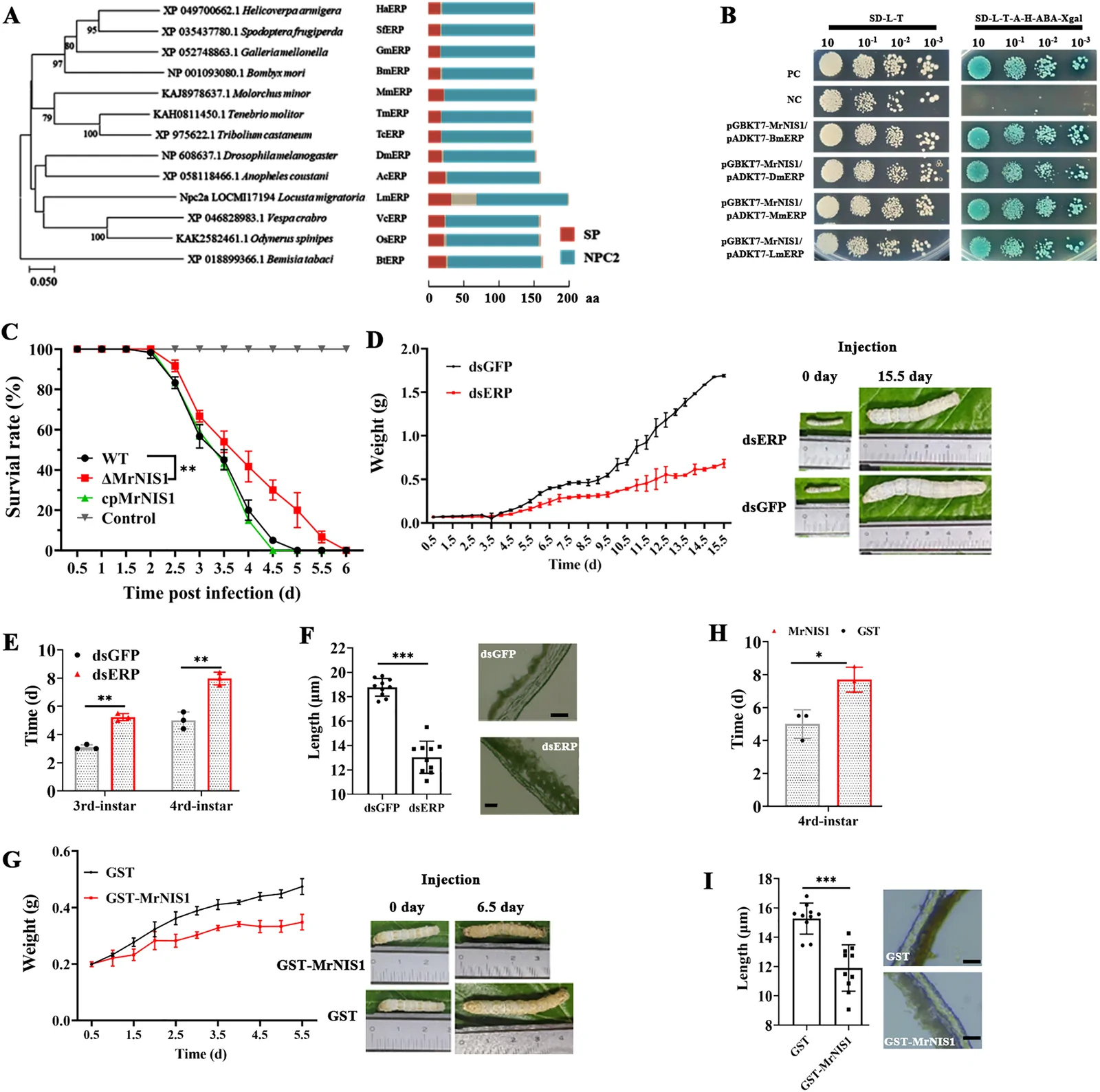
ERP is evolutionarily conserved across insects and interacts with MrNIS1. (A) Phylogenetic analysis of ERP homologs across insect orders. (B) Yeast two-hybrid assays showing interactions between MrNIS1 and ERP orthologs from *Bombyx mori* (BmERP), *Drosophila melanogaster* (DmERP), *Molorchus minor* (MmERP) and *Locusta migratoria* (LmERP). (C) Survival curves of *B. mori* larvae challenged with ΔMrNIS1. ^**^P < 0.01. (D–F) Effects of *BmERP* (dsERP) knockdown on *B. mori* larval weight change (D), larval instar duration (E) and cuticle thickness (F). 2-day-old third-instar larvae were injected with dsERP. Data are presented in (D and E) as mean ± SD from three independent experiments (n = 6 per group). Data are presented in (F) as mean ± SD from ten randomly selected larvae. Scale bar, 15 μm. (G–I) Effects of MrNIS1 on *B. mori* larval weight change (G), larval instar duration (H) and cuticle thickness (I). 2-day-old fourth-instar larvae were injected with 1 μg GST-MrNIS1. Data are presented in (G and H) as mean ± SD from three independent experiments (n = 6 per group). Data are presented in (I) as mean ± SD from ten randomly selected larvae. Scale bar, 15 μm. ^*^P < 0.05, ^**^P < 0.01, ^***^P < 0.001.

To validate the functional consequences of this interaction, we selected the *B. mori* as a model organism for detailed characterization. Infection with the ΔMrNIS1 mutant resulted in attenuated virulence compared to the wild-type strain, demonstrating that MrNIS1 is required for full virulence during infection of insect hosts (Fig 7C and Table B in S1 Appendix). RNA interference-mediated knockdown of ERP suppressed larval weight gain (Fig 7D and Figure D in S1 Appendix). We next examined developmental timing. ERP knockdown extended the larval instar duration (Fig 7E), indicating delayed molting progression. Histological analysis further revealed that the cuticle thickness of ERP-depleted larvae was markedly reduced with post-treatment compared to controls, confirming that ERP deficiency impairs cuticle synthesis and integrity (Fig 7F).

To determine whether MrNIS1 exerts similar effects through targeting *B. mori* ERP, we injected recombinant GST-MrNIS1 into the hemocoel of silkworm larvae. MrNIS1 administration reduced body weight gain rates and prolonged larval instar duration compared to GST-injected controls (Fig 7G and 7H). Consistent with these developmental defects, histological examination revealed decreased cuticle thickness in MrNIS1-treated larvae (Fig 7I).

Collectively, these findings demonstrate that the MrNIS1-ERP interaction mechanism is functionally conserved across insect hosts: MrNIS1 targets ERP to inhibit insect growth, extending developmental timing, and compromising cuticle synthesis.

### MrNIS1 as a portable virulence module in *B. bassiana*

Phylogenetic analysis revealed that MrNIS1 homologs are widely distributed throughout the genus *Metarhizium* but are conspicuously absent from other entomopathogenic fungal lineages (Figure E in S1 Appendix). To investigate whether the introduction of MrNIS1 could confer enhanced virulence to fungi lacking this effector, we heterologously expressed MrNIS1 in *B. bassiana*. Expression of MrNIS1 (Bb-MrNIS1) enhanced the virulence of *B. bassiana* against host insects compared to the wild-type strain (Fig 8A and Table B in S1 Appendix). Expression of a lysine 24 to arginine substitution mutant (Bb-MrNIS1^K24^) also increased fungal pathogenicity, albeit to a lesser extent than the wild-type MrNIS1. Both Bb-MrNIS1-GFP and Bb-MrNIS1^K24^-GFP were detected in host hemocytes during infection (Fig 8B). The accumulation of Bb-MrNIS1^K24^-GFP in hemocytes was lower than that of wild-type MrNIS1.

**Fig 8:**
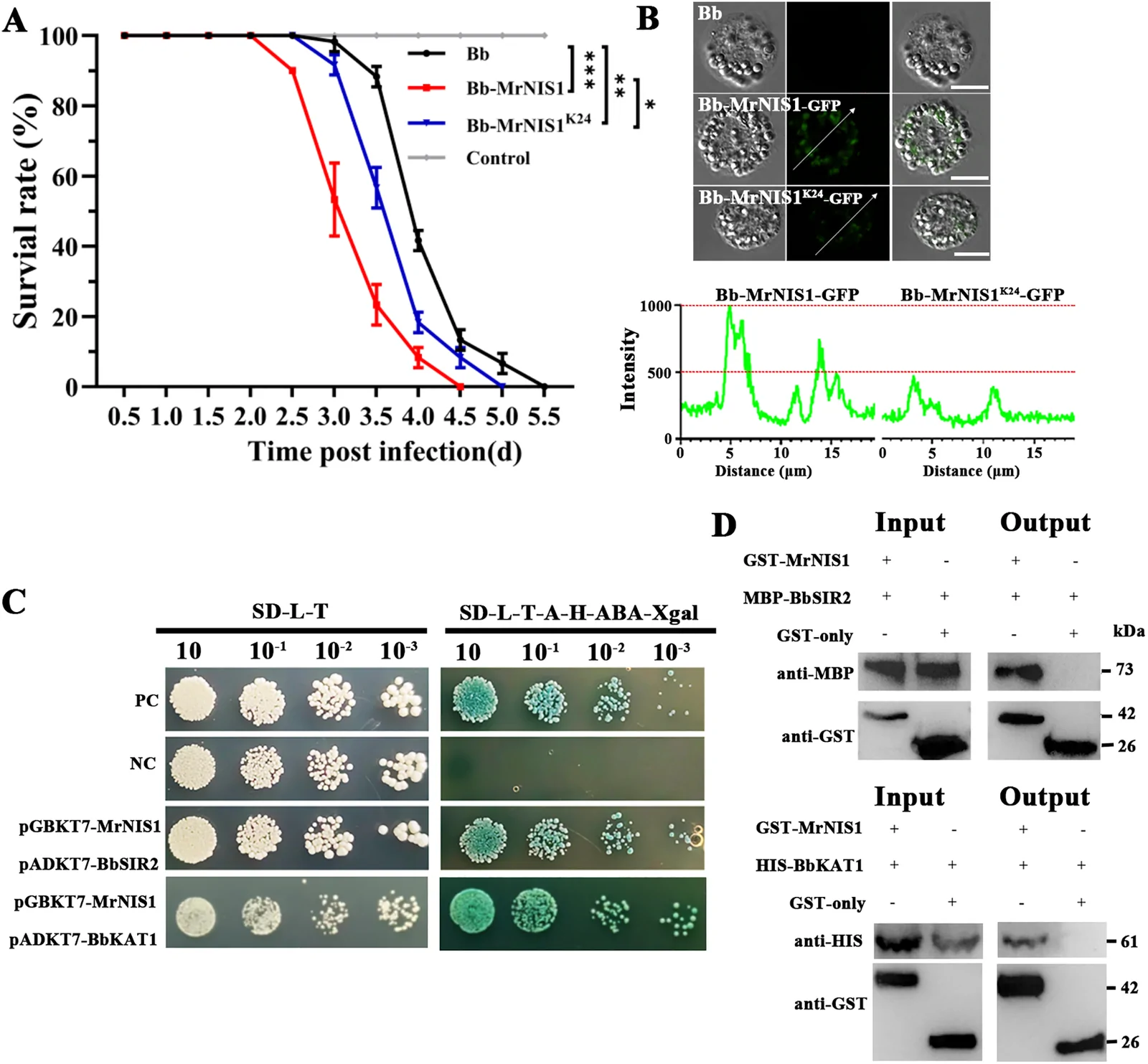
MrNIS1 interacts with the deacetylase BbSIR2 and acetyltransferase BbKAT1 in *B. bassiana.* (A) Survival curves of *G. mellonella* larvae infected by *B. bassiana* heterologously expressed with MrNIS1 or MrNIS1^K24^. ^*^P < 0.05, ^**^P < 0.01, ^***^P < 0.001. (B) Fluorescence microscopy of MrNIS1-GFP and MrNIS1^K24^-GFP localization in hemocytes from larvae infected by *B. bassiana* heterologously expressed with MrNIS1 or MrNIS1^K24^. Scale bar: 10 μm. (C) Yeast two-hybrid assays showing the interactions of MrNIS1 with the deacetylase BbSIR2 and the acetyltransferase BbKAT1. (D) GST pull-down assays showing the interaction of BbSIR2 and BbKAT1 with MrNIS1 *in vitro*.

Given that lysine residues are common targets for acetylation, we hypothesized that K24 might be post-translationally modified by acetylation in *B. bassiana*. Y2H and GST pull-down experiments demonstrated that MrNIS1 physically interacts with the *B. bassiana* deacetylase BbSIR2 and acetyltransferase BbKAT1 (Fig 8C and 8D), suggesting that MrNIS1 is subject to acetylation at K24 in this heterologous host. Heterologous expression demonstrates that the KAT1/SIR2 system is conserved across fungi, enabling MrNIS1 to function as a portable, acetylation-dependent virulence module that enhances pathogen efficacy in diverse genetic backgrounds.

## Discussion

Lysine acetylation has emerged as a pivotal post-translational modification that enables pathogen effectors to maintain functional competence during the transition between phylogenetically distant hosts. In the vascular wilt fungus *Fusarium oxysporum*, the effector FolSvp1 undergoes acetylation at K167, which not only ensures sufficient accumulation within fungal cells prior to secretion but also protects the protein from host-mediated proteasomal degradation after translocation into plant cells, thereby facilitating the manipulation of host immunity across kingdom boundaries [10]. Similarly, the reversible acetylation of the serine/arginine protein kinase FolSrpk1 at K304 governs its nucleocytoplasmic shuttling in response to oxidative stress, enabling the pathogen to fine-tune defense responses during host colonization [22]. In bacterial pathogenesis, YopJ family effectors such as HopZ5 and AvrBsT directly acetylate the conserved threonine residue T166 in the *Arabidopsis* immune regulator RIN4, mimicking phosphorylation-induced conformational changes to trigger effector-triggered immunity, illustrating the evolutionary arms race wherein pathogens deploy acetylation to manipulate host signaling while plants have evolved deacetylases to restore immune homeostasis [23]. Consistent with these findings, the present study demonstrates that acetylation of the *M. robertsii* effector MrNIS1 at K24 is essential for its stability and secretion in the fungal pathogen, while simultaneously protecting the protein from degradation during transit through the insect hemolymph. The observation that heterologous expression of MrNIS1 in *B. bassiana* yields an acetylated protein capable of enhancing fungal virulence further supports the notion that acetylation serves as a portable molecular signature that confers cross-species functionality to effector proteins. Collectively, these findings establish acetylation as a fundamental mechanism that enables successful cross-kingdom pathogenesis by preserving effector integrity across diverse host environments.

The functional antagonism between lysine acetylation and ubiquitination represents a critical regulatory switch that determines protein fate, as both modifications compete for the same ε-amino groups on lysine residues [24,25]. This competitive relationship has been demonstrated across diverse biological systems, where acetylation physically blocks ubiquitin conjugation, thereby preventing proteasome-mediated degradation and extending protein half-life. In *Salmonella typhimurium*, acetylation of the transcriptional regulator HilD at K297 by the acetyltransferase Pat enhances protein stability while concomitantly reducing DNA-binding affinity, demonstrating that acetylation serves as a molecular timer that maintains appropriate protein levels while modulating functional activity [26]. Similarly, in the rice blast fungus *M. oryzae*, the histone acetyltransferase MoHat1 acetylates autophagy-related proteins MoAtg3 and MoAtg9 at specific lysine residues, thereby regulating autophagic flux and maintaining protein homeostasis during infection-related morphogenesis [27]. Recent studies in *Verticillium dahliae* have further elucidated this mechanism, revealing that NatA-mediated acetylation of the molecular chaperone Hsp83 at K381 and K410 facilitates the formation of a co-chaperone complex with Sti1 and Hsp70 that is essential for protein quality control, whereas disruption of this modification leads to dissociation of the complex, enhanced protein ubiquitination, and subsequent apoptosis [28]. In the context of the present study, the finding that K24 acetylation is essential for MrNIS1 stability suggests a similar protective mechanism. Within *M. robertsii*, acetylation at K24 likely protects MrNIS1 from premature degradation during biosynthesis and trafficking to the vacuole, ensuring that the effector accumulates to levels sufficient for secretion. Furthermore, the reduced accumulation of MrNIS1^K24^ in host hemocytes compared to the wild-type protein demonstrates that acetylation continues to provide protection against host degradative activities in the hostile environment of the insect hemolymph.

Previous study demonstrated that MrSIR2 and MrKAT1 physically associate with target proteins and exert opposing enzymatic activities to modulate protein function [18]. In the present study, we identified both enzymes as physical interactors of MrNIS1, and mass spectrometry confirmed K24 acetylation *in vivo*. These findings raise the possibility that MrNIS1 acetylation is subject to reversible enzymatic control by MrKAT1 and MrSIR2, analogous to their antagonistic regulation of other substrates [18]. Direct evidence for such a switch operating on MrNIS1 *in vivo* is currently lacking, and future studies employing acetylation-site-specific antibodies and *in vitro* enzyme assays will be necessary to test this model.

The melanization immune response represents one of the most ancient and conserved defense mechanisms in arthropods, wherein the sequential activation of extracellular serine protease cascades culminates in the proteolytic conversion of PPO to active PO, thereby triggering the synthesis of cytotoxic melanin that encapsulates and eliminates invading pathogens [29]. The critical roles of clip-domain serine proteases in PPO activation have been demonstrated across diverse insect species, including MP2 in *D. melanogaster*, and in *Ostrinia furnacalis* [30,31]*.* However, pathogens have evolved sophisticated strategies to subvert this host defense mechanism. For instance, the rice orange leaf phytoplasma effector SRP1, suppresses the melanization response in the leafhopper vector by binding to the serine protease SP2, thereby obstructing the cleavage of PPO2 and inhibiting PO activity [19]. Similarly, entomopathogenic fungi including *Beauveria* and *Metarhizium* species actively suppress host melanization through the secretion of secondary metabolites such as oosporein and destruxins, which inhibit PO activity and downregulate PPO gene expression [32,33]. The metalloproteinase MrM35-4 from *M. robertsii* further exemplifies this immune evasion strategy by cleaving PPO at non-activation sites, thereby preventing the generation of functional PO [34]. In the present study, we demonstrate that the *M. robertsii* effector MrNIS1 employs a distinct yet convergent mechanism to suppress host melanization by competitively binding to the serine protease GmSPS, thereby physically blocking the interaction between GmSPS and its substrate GmPPO. Moreover, *GmSPS* transcript levels declined rapidly following fungal entry into the hemocoel. This downregulation may reflect pathogen-induced transcriptional suppression of host immune genes**,** feedback attenuation resulting from MrNIS1-mediated GmSPS sequestration, or progressive tissue damage during late infection. Diminished GmSPS availability would further impair PPO activation, synergizing with the direct inhibitory effect of MrNIS1 to suppress melanization. In agreement with the predicted role of MrNIS1 in melanization suppression, larvae infected with ΔMrNIS1 exhibited elevated PO activity and enhanced melanization compared to WT-infected controls, providing compelling *in vivo* evidence that MrNIS1 functions as a virulence factor that attenuates host melanization immunity.

NPC2 family proteins, including ERPs, serve as essential mediators of cholesterol trafficking in insects, a function that is fundamentally linked to ecdysteroid biosynthesis and developmental progression [20]. In *D. melanogaster*, NPC2a and NPC2b function redundantly in intracellular cholesterol trafficking, and double mutants exhibit larval lethality, demonstrating that the primary developmental defect stems from impaired ecdysteroid biosynthesis [35]. Knockdown of NPC1, works synergistically with NPC2, results in cholesterol accumulation and developmental arrest at the larval molting stage, underscoring the critical role of cholesterol transport in insect metamorphosis [36]. The functional importance of NPC2 proteins extends beyond cholesterol trafficking to directly influence cuticle formation, as ecdysteroids regulate the expression of genes involved in chitin synthesis and cuticle sclerotization [37]. The phylogenetic conservation of ERP across Lepidoptera, Diptera, Coleoptera, and Orthoptera, combined with the demonstration that MrNIS1 binds to ERP orthologs from multiple insect species, suggests that the MrNIS1-ERP interaction represents a broadly applicable virulence strategy. By targeting ERP, MrNIS1 likely disrupts cholesterol trafficking and consequently impairs ecdysteroid biosynthesis, leading to the observed defects in larval growth, extended instar duration, and reduced cuticle thickness. This mechanism parallels the function of other entomopathogenic fungal effectors that manipulate host hormone signaling, but distinctively targets the upstream cholesterol transport machinery rather than directly interfering with hormone receptors or signaling cascades [38].

In conclusion, we identify a dual-targeting strategy in *M. robertsii* mediated by the acetylated effector MrNIS1. By competitively binding GmSPS, MrNIS1 blocks PPO activation to suppress host immunity, while simultaneously targeting the conserved transporter GmERP to disrupt larval development (Fig 9). The observation that MrNIS1 enhances virulence upon heterologous expression in *B. bassiana* identifies this effector as a portable virulence module with broad functional potential. Our findings establish that post-translational acetylation is a critical requirement for effector stability during cross-kingdom transit, enabling the precise targeting of evolutionarily conserved host nodes. This regulatory mechanism provides a molecular basis for the strategic engineering of high-efficacy microbial biocontrol agents.

**Fig 9:**
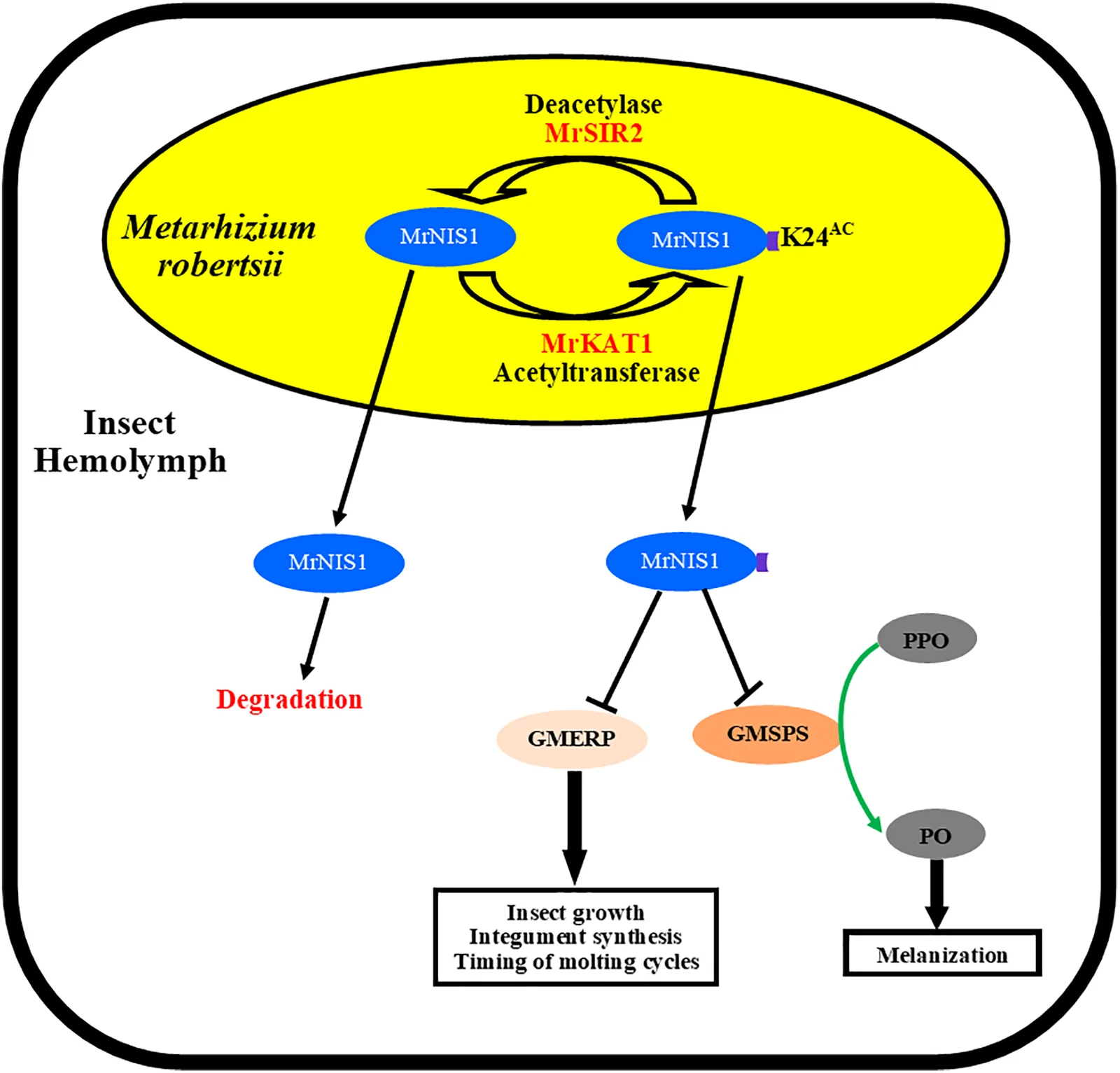
Proposed model for how the acetylated effector MrNIS1 disrupts development and inhibits the melanization immune response in the insect hemolymph. A dual-targeting virulence strategy in *M. robertsii* wherein the acetylated effector MrNIS1 simultaneously suppresses insect immunity by blocking PPO activation via competitive GmSPS binding and disrupts development by targeting the conserved cholesterol transporter GmERP.

## Materials and methods

### Fungal culture and insect rearing

The WT strains ARSEF 23 of *M. robertsii* and ARSEF 2860 of *B. bassiana* were used in this study [39,40]. Fungal strains were cultured on potato dextrose agar (PDA; BD Difco, BD 213300) at 25 °C for two weeks in the dark, then the conidial suspension from this culture used for *Agrobacterium*-mediated transformation. *G. mellonella* (greater wax moth; Kaide Ruixin, Tianjin, China) and *B. mori* (silkworm moth; Nanning Yongzhou Sericulture, Nanning, China) were maintained based on a previous study [17,41].

### Yeast two-hybrid library screening and interaction assays

To identify proteins interacting with MrNIS1, the coding sequence lacking the N-terminal signal peptide was cloned into pGBKT7 and transformed into Y2HGold yeast for screening against *M. robertsii* and *G. mellonella* cDNA libraries. Positive clones were selected and verified by sequencing. Similarly, full-length GmSPS was used as bait to screen the *G. mellonella* cDNA library for identifying interacting partners. For validation, candidate genes were cloned into pGADT7 and co-transformed with respective bait constructs into Y2HGold, with interactions assessed using synthetic dropout-Leu-Trp medium and Leu-Trp-His-Ade/X-α-gal/AbA plates as previous report [18]. Empty vectors and the pGADT7-T/PGBKT7–53 antigen pair served as negative and positive controls, respectively.

### Pull down and competitive pull down

Protein interactions were confirmed using the GST or HIS pull-down system. Recombinant fusion proteins were expressed in *E. coli* BL21 (DE3; TransGen, Beijing, China) and purified using affinity chromatography. For GST-MrNIS1 pull-down assays, GST-tagged bait proteins were immobilized on *ProteinIso* GST Resin (TransGen, Beijing, China) by resuspending the resin in binding buffer and incubating at 4°C for 2 h. The column was then equilibrated with 10 column volumes of wash buffer to remove unbound proteins. Purified prey proteins (MBP-MrSIR2, HIS-MrKAT1, HIS-GmSPS, MBP-GmERP, MBP-BbSIR2, and HIS-BbKAT1) were diluted and filtered through 0.45 μm membranes, loaded onto the GST-MrNIS1-bound resin. Following incubation at 4°C for 2 h, the column was washed with 10 column volumes of ice-cold wash buffer to eliminate non-specific binding. Bound protein complexes were eluted using 0.1 M glycine-HCl (pH 2.5-3.0) and immediately neutralized with 1 M Tris-HCl (pH 9.0). Eluted samples were then analyzed by immunoblotting with anti-GST, anti-MBP, and anti-His antibodies (Beyotime Biotechnology, Shanghai, China). Negative controls included GST alone incubated with individual prey proteins. For the GmSPS-GmPPO interaction, HIS-GmSPS was immobilized on *ProteinIso* Ni-NTA Resin (TransGen, Beijing, China) and incubated with MBP-GmPPO. Following washing, bound complexes were eluted and detected by immunoblotting using anti-HIS and anti-MBP antibodies as described above.

Competitive binding assays were performed to evaluate the different binding affinities among MrNIS1, GmSPS, and GmPPO. HIS-GmSPS was immobilized on *ProteinIso* Ni-NTA Resin and incubating at 4°C for 2 h, followed by equilibrating with wash buffer to remove unbound proteins. The resin-bound HIS-GmSPS was then incubated with gradient concentrations of GST-MrNIS1 together with a constant amount of MBP-GmPPO at 4°C for 2 h to assess the dose-dependent effect of GST-MrNIS1 on the recruitment of MBP-GmPPO. Unbound proteins were removed with wash buffer, the resulting complexes were captured using glycine-HCl and Tris-HCl. The bound proteins were separated and detected as described above.

### Acetylome profiling and analysis

*M. robertsii* was cultured on Potato Dextrose Agar for two weeks in the dark. Mycelia were lysed in SDS buffer containing protease inhibitors and 10 mM DTT, homogenized (60 Hz, 2 min), and centrifuged (25,000 × g, 15 min, 4°C). Proteins were reduced (10 mM DTT, 56°C, 1 h), alkylated (55 mM iodoacetamide, 45 min, dark), precipitated with cold acetone (-20°C, 30 min), and quantified by Bradford assay. Proteins (30 μg) were resolved by 8% SDS-PAGE, Coomassie-stained, and subjected to in-gel trypsin digestion (37°C, overnight). Peptides were extracted, lyophilized, and enriched using anti-acetyllysine antibody-conjugated beads (CST, 4°C, overnight). After washing and elution (0.15% TFA), peptides were analyzed by nanoLC-MS/MS using a Q Exactive HF-X mass spectrometer. Separation was performed on a C18 column (75 μm × 25 cm) with a 60-min gradient (5–35% ACN). Full MS scans were acquired at 60,000 resolution (m/z 350–1,500), followed by HCD-MS/MS (15,000 resolution, NCE 30) of the top 30 precursors (charge 2–6, dynamic exclusion 30 s). Data were searched with MaxQuant (v.1.6.17.0) against the *M. robertsii* UniProt database (FDR 1%).

### Gene deletion, complementation, and site-directed mutagenesis

Gene deletion mutants were generated by *Agrobacterium tumefaciens*-mediated transformation (ATMT). The 5’- and 3’-flanking regions (~1.1 kb each) of MrNIS1 were amplified and ligated into pDHt-bar (conferring glufosinate resistance) flanking the bar cassette. The resulting knockout vector was transformed into *A. tumefaciens* AGL1, followed by co-cultivation with *M. robertsii* conidia on induction medium. Gene deletion was verified by PCR analysis. For complementation and site-directed mutagenesis, the full-length MrNIS1 coding sequence (including native promoter) or its lysine-to-arginine mutants (∆MrNIS1/MrNIS1^K24^ and ∆MrNIS1/MrNIS1^K120^) was inserted into pDHt-ben (conveying benomyl resistance) and the resulting plasmids were introduced into the ΔMrNIS1 mutant via ATMT. For GFP tagging, MrNIS1, MrNIS1^K24^ and MrNIS1^K120^ were fused to GFP at the C-terminus and expressed in the ΔMrNIS1 background under the native promoter. For heterologous expression in *B. bassiana*, MrNIS1 and MrNIS1^K24^-GFP driven by the native promoter were cloned into pDHt-ben and introduced into *B. bassiana* via ATMT. Transformants were selected and verified by PCR.

### Western blotting and immunoprecipitation

*M. robertsii* strains were cultured in potato dextrose liquid medium at 25°C with shaking (150 rpm) for 3 d. Culture supernatants were harvested by centrifugation (8,000 × g, 10 min, 4°C) and filtration (0.22 μm). Protein extracts were incubated with anti-GFP agarose beads (Beyotime Biotechnology, Shanghai, China) at 4°C for 4 h. After washing, immunoprecipitates were subjected to immunoblotting with anti- GFP antibody (Abmart, Shanghai, China) and pan anti-acetyl-lysine antibody (PTM Biolabs, Hangzhou, China). Signals were detected using ECL reagent and captured by ChemiDoc XRS+ system (Bio-Rad).

### Insect bioassays

Fifth-instar *G. mellonella* and *B. mori* larvae were used to assess the virulence of fungal strains. Conidia were administered by immersing the larvae into a 10^6^ conidia mL^-1^ suspension. Larvae were immersed in the conidial suspension for 30 s, blotted dry on sterile filter paper, and transferred to sterile plastic containers (20 larvae per container) with artificial diet at 25°C and 70% relative humidity. Tween-80 treated groups were used as control. For each treatment, three biological replicates were performed (n = 60). Additionally, hemolymph was collected from infected larvae for further analysis. For gene expression analysis, total RNA was extracted from infected larvae hemolymph using TRIzol reagent for quantitative RT-PCR analysis of *GmSPS* and *GmERP* transcript levels.

### *Ex vivo* acetylation stability assay

GFP-tagged MrNIS1^K24^, MrNIS1^K120^ and MrNIS1 were purified from *M. robertsii* mycelia using anti-GFP agarose beads. Equal amounts of purified proteins were incubated with *G. mellonella* hemolymph (diluted 1:10 in PBS) at 25°C for 4 h. Proteins were then precipitated with 15% TCA and subjected to immunoblotting with anti-GFP antibody to monitor protein stability.

### RNA interference

DsRNAs targeting *SPS*, *ERP*, or *GFP* (control) genes were synthesized using the MEGAscript RNAi Kit (Invitrogen, Massachusetts, USA). To ensure RNAi stability and efficiency in lepidopteran insects, we employed our previously established core-shell polymeric nanostructure (PRn)-mediated dsRNA delivery system [42]. For RNAi-mediated knockdown, different delivery methods were employed based on insect species. *Bombyx mori* larvae were orally administered 10 μL of PRn@dsRNA complex (containing 1 μg dsRNA), and *G. mellonella*. larvae were injected with 5 μL of PRn@dsRNA complex (containing 0.5 μg dsRNA) into the hemocoel via the abdominal proleg using a microsyringe. Control groups received equivalent doses of dsGFP complex.

### Phenoloxidase (PO) activity assay

Hemolymph was collected from larvae at designated time points post-treated by piercing the abdominal proleg and immediately diluted in ice-cold 10 mM Tris-HCl buffer (pH 8.0) and centrifuged at 4,000 × g for 30 min at 4°C to pellet hemocytes. The supernatant (20 μL) was then mixed with 100 μL of 1 mM dopamine (dissolved in 10 mM Tris-HCl, pH 8.0) in a 96-well plate. Following incubation at room temperature for 5 min, the absorbance at 490 nm (A_490_) was monitored every 10 min using a microplate reader (Synergy HTX; BioTek, USA). PO activity was calculated using the formula provided in the phenoloxidase assay kit (Beyotime Biotechnology, Shanghai, China). Activity assays were independently repeated using hemolymph samples from six biological replicates.

### Fluorescence microscopy and line-scan intensity analysis

Confocal images were acquired on a Zeiss LSM 880 confocal microscope (63 × oil-immersion objective, NA 1.40). All comparative images were captured using identical acquisition parameters (laser power, gain, pinhole, and pixel dwell time). Line-scan fluorescence intensity profiles were generated using the Plot Profile tool in ImageJ/Fiji along the indicated positions (white arrows).

### Statistical analysis

All data presented in this paper are derived from a minimum of three biological replicates. Statistical analyses were performed using GraphPad Prism 9.0.0. For multiple group comparisons, one-way analysis of variance (ANOVA) was conducted, followed by Tukey’s multiple comparison test to evaluate significant differences among different experimental groups or treatments. Statistical significance was defined as *P* < 0.05.

## Supporting information

S1 Appendix Figure A. Transcriptional profiling of *MrNLS1* at different infection stages. ^***^P < 0.001. Figure B. Knockdown of *GmSPS* and *GmERP* expression analyzed by qPCR. Lavae injected with 0.5 μg dsRNA at 36 h and 48 h were collected for qPCR analysis. The green fluorescent protein (ACY56286) gene was used as the control. ^***^P < 0.001. Figure C. Predicted structure of the GmSPS-MrNIS1 complex generated by ZDOCK server. GmSPS is shown in red and MrNIS1 in blue. Figure D. Knockdown of *BmERP* expression analyzed by qPCR. Lavae orally administered 1 μg dsRNA at 36 h and 48 h were collected for qPCR analysis. The green fluorescent protein (ACY56286) gene was used as the control. ^**^P < 0.01. Figure E. Phylogenetic analysis of NIS1 across diverse organisms. Phylogenetic tree of selected NIS1 homologs reconstructed as previous report [14]. MrNLS1 showed in the Figure has been reported in our previous study [17]. Table A. Interacting proteins screening of MrNIS1 from *Metarhizium robertsii*. Table B. Abbott-corrected median lethal time (LT_50_) values (mean ± SD) for survival assays. Table C. Interacting proteins screening of MrNIS1 from the *Galleria mellonella*. Table D. Interacting proteins screening of GmSPS.
(DOCX)
