# Supplementary material for: Acetylation enables a dual-targeting fungal effector to inhibit insect immunity and development
Source: PLoS Pathog. 2026 Oct 5;22(10):e1014680. doi: 10.1371/journal.ppat.1014680 (PMC13660978; doi:10.1371/journal.ppat.1014680)
Supplement: Supplementary file 1 [file ppat.1014680.s001.docx]

S1 Appendix of

**Acetylation enables a dual-targeting fungal effector to inhibit insect immunity and development**

Yulong Wang^1,†,*^, Ying Dong^1,†^, Yang Zhang^1^, Siying Wu^1^, Deshui Yu^1,2^, Guang Yang^1^, Shuqian Gao^1^, Yang Yang^1^, Shihao Dong^1^, Li Qin^1^, Bo Huang^1,*^

^1^Anhui Provincial Key Laboratory of Biological Control, Anhui Agricultural University, Hefei 230036, China

^2^Hefei Forestry Protection Center, Hefei 230036, China

^†^These authors contributed equally: Yulong Wang, Ying Dong

^*^ Correspondence: Bo Huang [bhuang@ahau.edu.cn](mailto:bhuang@ahau.edu.cn); Yulong Wang [wyl2019@ahau.edu.cn](mailto:wyl2019@ahau.edu.cn)

**This PDF file includes:**

A to E Figures

A to D Tables

References SI

Figures

Figure A. Transcriptional profiling of *MrNLS1* at different infection stages. ^***^*P* < 0.001.

Figure B. Knockdown of *GmSPS* and *GmERP* expression analyzed by qPCR. Lavae injected with 0.5 μg dsRNA at 36 h and 48 h were collected for qPCR analysis. The green fluorescent protein (ACY56286) gene was used as the control. ^***^*P* < 0.001.

Figure C. Predicted structure of the GmSPS-MrNIS1 complex generated by ZDOCK server. GmSPS is shown in red and MrNIS1 in blue.

Figure D. Knockdown of *BmERP* expression analyzed by qPCR. Lavae orally administered 1 μg dsRNA at 36 h and 48 h were collected for qPCR analysis. The green fluorescent protein (ACY56286) gene was used as the control. ^**^*P* < 0.01.

Figure E. Phylogenetic analysis of NIS1 across diverse organisms. Phylogenetic tree of selected NIS1 homologs reconstructed as previous report (1) MrNLS1 showed in the Figure has been reported in our previous study (2).

Tables

Table A. Interacting proteins screening of MrNIS1 from *Metarhizium robertsii*.

| Table S1 Interacting proteins screening of MrNIS1 from *Metarhizium robertsii* | |
| --- | --- |
| Gene ID | Annotation |
| XP_007823864.1 | Glyceraldehyde/Erythrose phosphate dehydrogenase family |
| XP_007825148.1 | uncharacterized protein |
| XP_007818254.1 | NAD-dependent histone deacetylase, silent information regulator Sir2 |
| XP_007821503.1 | ATP synthase beta chain |
| XP_007819986.2 | translation elongation factor 1-alpha |
| XP_007826457.1 | S1/P1 Nuclease |
| XP_007821864.1 | alkaline serine protease P32 |
| XP_007817815.1 | polyketide synthase 3 |
| XP_007823745.2 | gamma-actin |
| XP_011411800.1 | uncharacterized protein |
| XP_007821750.1 | uncharacterized protein |
| XP_007825938.1 | Fungal fucose-specific lectin |
| XP_007817563.1 | H3 K56 histone acetylation protein KAT11 |

| Table B Abbott-corrected median lethal time (LT_50_) values (mean ± SD) for survival assays. | |
| --- | --- |
| Strains | Mean LT₅₀ ± SD (d) |
| LT_50_ values of *Galleria mellonella* larvae infected by different strains | |
| WT | 3.20 ± 0.04 |
| ∆MrNIS1 | 4.47 ± 0.12 |
| ∆MrNIS1/MrNIS1^K24^ | 3.68 ± 0.21 |
| ∆MrNIS1/MrNIS1^K120^ | 3.30 ± 0.01 |
| cpΔMrNIS | 3.38 ± 0.04 |
| LT_50_ values of *G. mellonella* larvae challenged with *Metarhizium robertsii* after RNA interference targeting *GmERP* | |
| WT-dsGFP | 3.45 ± 0.08 |
| WT-dsERP | 3.07 ± 0.06 |
| LT_50_ values of *G. mellonella* larvae challenged with *M. robertsii* after RNA interference targeting *GmSPS* | |
| WT-dsGFP | 3.44 ± 0.03 |
| WT-dsSPS | 3.08 ± 0.05 |
| LT_50_ values of *Bombyx mori* larvae challenged with ΔMrNIS1 | |
| WT | 3.25 ± 0.25 |
| ΔMrNIS | 3.67 ± 0.29 |
| cpMrNIS | 3.28 ± 0.10 |
| LT_50_ values of *G. mellonella* larvae infected by *Beauveria bassiana* heterologously expressed with MrNIS1 or MrNIS1^K24^ | |
| Bb | 3.85 ± 0.04 |
| Bb-MrNIS | 3.17 ± 0.11 |
| Bb-MrNIS1^K24^ | 3.48 ± 0.06 |

Table C. Interacting proteins screening of MrNIS1 from the *Galleria mellonella*.

| Table S3 Interacting proteins screening of MrNIS1 from the *Galleria mellonella* | |
| --- | --- |
| Gene ID | Annotation |
| XP_026760882.2 | fibroin heavy chain |
| XP_026751084.2 | antichymotrypsin-2 isoform X9 |
| XP_052748863.1 | ecdysteroid-regulated 16 kDa protein |
| XP_031766532.2 | serine protease snake-like |
| XP_052757976.1 | probable ATP-dependent RNA helicase Dbp73D isoform X2 |

Table D. Interacting proteins screening of GmSPS.

| Table S4 Interacting proteins screening of GmSPS | |
| --- | --- |
| Gene ID | Annotation |
| XP_052748312.1 | nuclease SbcCD subunit C-like |
| XP_026762851.1 | WW domain-binding protein 11 |
| XP_052758720.1 | cholinephosphotransferase 1 |
| XP_052756470.1 | uncharacterized protein |
| XP_052753939.1 | kunitz-like toxin PcKuz3 |
| XP_026760150.2 | actin |
| XP_026755528.1 | serine protease gd-like |
| XP_026751492.2 | antichymotrypsin-2-like isoform X4 |
| XP_026752942.1 | mitotic spindle assembly checkpoint protein MAD2A |
| XP_052748312.1 | nuclease SbcCD subunit C-like |
| XP_026755528.1 | serine protease gd-like |
| XP_031766766.1 | E3 ubiquitin-protein ligase cblA-like |
| XP_052756923.1 | arylphorin-like |
| XP_026764452.2 | filamin-A |
| XP_026763508.2 | alpha-amylase 2-like isoform X1 |
| XP_026753329.1 | chitin deacetylase 8-like |
| XP_052751666.1 | phenoloxidase |

**SI References**

Sample References:

1 Irieda, H. *et al.* Conserved fungal effector suppresses PAMP-triggered immunity by targeting plant immune kinases. *Proceedings of the National Academy of Sciences of the United States of America* **116**, 496-505 (2019). https://doi.org/10.1073/pnas.1807297116
2 Wang, Y. L. *et al.* Conserved fungal effector NLS1 suppresses Lepidoptera insect immunity by targeting the host defense protein Hdd11. *Insect Science* **32**, 1298-1310 (2025). https://doi.org/10.1111/1744-7917.13454
